# 

**Published:** 1875-02

**Authors:** 


*To our Subscribers.*

☞ Please remember that the Southern Publishing Company have contracted for the publication of the RECORD for the year 1875. The publishers have pledged themselves to issue the RECORD between the 1st and 15th of each month, and in the style and appearance of the present number. The Southern Publishing Company will have charge of the subscription and mailing books, and will be responsible for the prompt and thorough mailing of the RECORD to each subscriber.

All amounts for subscription for the year 1875 must be forwarded to the Southern Publishing Company by Post-office Money Order or Registered Letter, and the Company pledge themselves to acknowledge receipt of subscriptions in the following issue of the RECORD.

Our friends will please inform the publishers or principal editor of any failure in the mailing department, or omission of proper acknowledgments of the reception of subscriptions. Any errors that may occur will be promptly remedied.

Our friends who may send in the name of a new subscriber, and who desire to get the premium offered (Powell's Pocket Formulary), will please signify the same to the publishers, in order that the premium may be forwarded to them at once. Give us your prompt and unwavering support, friends.

☞ All due for 1873 and 1874 can be directed to Dr. T. S. Powell, or Powell & Goldsmith, as heretofore.

☞ We shall print several hundred extra copies of the January and February numbers of the RECORD, that all new subscribers who send in their names between this and the tenth of March, may have the volume for 1875 complete. Be certain to be in time.

☞ All communications other than of a business character must be addressed to Dr. Thos. S. Powell, 82 Pryor street.

☞ Write your name, post-office, county and State *plainly*. Be sure to say to which post-office you wish the RECORD sent, and always date your letters. We receive many well-written letters, but doubt even the authors being able to decipher their signatures.

☞ Send money by Check, Postal Order or Registered Letter. We are willing to endorse for the honesty of our subscribers, but cannot be responsible for the irregularity of the mails.

☞ We hope our friends, one and all, will rally to the support of the RECORD. Send the publishers three dollars, the subscription fee, and at least one new subscriber. Send to the principal editor your communications, plainly and carefully written on only one side of the paper.

### LIST OF CASH PAYMENTS SINCE JANUARY NUMBER.

| | |
|---|---|
| Dr. O. Gregory, North Carolina | $3 00 |
| Dr. J. . Baker, Louisiana | 3 00 |
| Dr. L. D. Johnson, Georgia | 3 00 |
| *Dr. C. H. Smith, Georgia | 2 50 |
| Dr. E. A. Anderson, North Carolina | 3 00 |
| Dr. J. W. Etheridge, Mississippi | 3 00 |
| Dr. J. W. Bennett. North Carolina | 3 00 |
| Dr E. T. Easley, Texas | 3 00 |
| Dr Thos. S. Parham, Louisiana | 3 00 |
| Dr. D. B. Hamilton, Louisiana | 3 00 |
| Dr. Wm. B. Watford, North Carolina | 3 00 |
| Dr. Lockwood Alison. Louisiana | 3 00 |
| Dr. J. L. Martin, Oregon | 3 00 |
| Dr. N. B. Richards. Illinois | 3 00 |
| Dr. A. C. Helm. Oregon | 3 00 |
| Dr. H. Logan. Oregon | 3 00 |
| Dr. J. C. Knox, Alabama | 3 00 |
| Dr. Alfred H. Ketchum, Texas | 3 00 |
| Dr. A. P. Harris, Mississippi | 3 00 |
| Dr. M. J. Laster, Mississippi | 3 00 |
| Dr. J. I. Grover. Georgia | 3 00 |
| Dr. Wm. H. Welson, Georgia | 3 00 |
| Dr. J. McQ. Stansill, North Carolina | 3 00 |
| Dr. R. S. Hallsey. North Carolina | 3 00 |
| Dr. A. R. Kilpatrick, Texas | 30 |
| Dr. S. M. Hogan, Alabama | 50 |
| Dr. B. F. Darnell. Alabama | 50 |
| Dr. Samuel H. Hill, Alabama | 3 00 |
| *Dr. E. N. Cushion, Colorado Territory | 2 50 |
| Dr. J. M. Williamson, Mississippi | 1 00 |
| Dr. P. W. Callehan, Louisiana | 3 00 |
| *Dr. T. J. Burns, Mississippi | 2 50 |
| *Dr. Robt. Kells. Mississippi | 2 50 |
| Dr. H. Kelly, Mississippi | 2 65 |
| Dr. T. J. Jones, Georgia | 3 00 |
| *Dr. N. G. West. Virginia | 2 50 |
| *Dr. R. H. Edwards, Virginia | 2 50 |
| Dr. A. R. Tharp. North Carolina | 3 00 |
| Dr. C. B. Galloway. Mississippi | 3 00 |
| Dr. R Bevier, Mississippi | 50 |
| Dr. W. A. Cusick, Oregon | 3 00 |
| *Dr. L. Farrow, New Jersey | 2 50 |
| *Dr. Joseph Hedges, New Jersey | 2 50 |
| *Dr. Joseph P. Couse, New Jersey | 2 50 |
| *Dr. B. F. Powell. Alabama | 2 50 |
| *Dr. J. R. Hicks, Mississippi | 2 50 |
| *Dr. E. T. Henry. Mississippi | 2 50 |
| *Dr. Geo. A. Foote, North Carolina | 2 50 |
| Dr. T. C. McGuffey, Louisiana | 3 00 |
| Dr. W. G. Knox. Louisiana | 3 00 |
| *Dr. M. R. Harrison. Mississippi | 2 50 |
| Dr. Jno. H. Weir, Illinois | 3 00 |

*Please remit fifty cents.

### COLLABORATORS AND CONTRIBUTORS.

**GEORGIA.**

James A. Long, M.D.
W. L. M. Harris, M.D.
H. L Battle, M.D.
W. C. Musgrove, M D
L B. Bouchelle, M.D.
Thomas Burdell, M.D.
E. H. W. Hunter, M.D.
V. S Hitt, M.D.
J. E. Pope, M.D.
J. C. Wisdom, M.D.
W. W. Leake, M.D.
S. G. White, M.D.
W. H. Hall, M.D.
G. D. Case, M.D.
W. T. Grant, M.D.
J. M. Tules, M.D.
J. N. Cheney, M.D.
T. M. Shaw, M.D.
T. S. Mitchell, M.D.
H. B. Lee, M.D.
C. B. Nottingham, M.D.
W. L. Kirkpatrick, M.D.

**KENTUCKY.**

W. S. Taylor, M.D.
B. W. Stone, M.D.
Charles Kearns, M.D.

**TEXAS.**

S. M. Welch, M.D.
T. J. Heard, M.D.
L. R. Lincecum, M.D.

**TENNESSEE.**

J. D. Tucker, M.D.

F. A. Ramsey, M.D.

J. W. Hayne, M.D.

F. K. Bailey, M.D.

**WEST VIRGINIA.**

N. D. Tobey, M.D.

**ALABAMA.**

J. C. Knox, M.D.
Geo. F. Taylor, M.D.
J. B. Barnett, M.D.
S. M. Hogan, M.D.
J. T. Searcy, M.D.
A. C. Edwards, M D.
M. W. Morton, M.D.
J. D. Rush, M.D.
J. J. Grace, M.D.

**NORTH CAROLINA.**

J. B. Hughes, M.D.
S. S. Satchwell, M.D.
A. B. Pierce, M.D.
H. Kelly, M.D.
Geo. A. Foote, M.D.
E. A. Anderson, M.D.
H. T. Bahnson, M.D.
John W. Booth, M.D.
R. S. Hallsey, M.D.

**SOUTH CAROLINA.**

E. T. McSwain, M.D.
G. M. Rivers, M.D.

**OHIO.**

J. Q. A. Hudson, M.D.
C. H. Tidd, M.D.
J. C. Bishop, M.D.
W. P. Wells, M.D.

**NEW YORK.**

Ely Van DeWarker, M. D.
C. C. F. Gay, M.D.
J. S. Bailey, M.D.

**COLORADO.**

Wm. R. Whitehead, M.D.

**VIRGINIA.**

F. Horner, Jr., M.D.
R. J. Preston, M.D.
J. S. Wharton, M.D.
Sam'l P. Ripley, M.D.
E. A. Drewry, M.D.
Rob B. Tunstall, M.D.
W. F. Barr, M.D.
L. B. Anderson, M.D.
J. N. Upshur, M.D.
B. P. Reese, M.D.

**MISSISSIPPI.**

C. B. Gallaway, M.D.
Edward Lea, M.D.
S. V. D. Hill, M.D.
E. T. Henry, M.D.
T. P. Lockwood, M.D.
Robert Kells, M.D.
W. L. Lipscomb, M.D.
W. T. Beall, M.D.
A. G. Smythe, M.D.
J. M. Lewis, M.D.
J. H. Payne, M.D.
T. H. Mayo, M.D.
E. Cutter, M.D.

**ARKANSAS.**

A. D. Hodge, M.D.
J. K. Hodge, M.D.
A. W. Hobson, M.D.
C. D. Hodge, M.D.

**INDIANA.**

G. A. Dyer, M.D.
A. Patton, M.D.
Robert Robson, M.D.

**ILLINOIS.**

John H. Weir, M.D.

**OREGON.**

W. M. Cusick, M.D.

[SUPPLEMENT.]

### PUBLISHERS' NOTICE TO SUBSCRIBERS.

---

The publishers intend to carry their contract to publish the RECORD into full effect. The delay in the appearance both of the January and February numbers has been caused by unavoidable circumstances. The publishers could have given the RECORD to its readers on time had it not been for the fact that they (and the editors) determined to print it on the finest rose-tinted book paper, a full year's supply of which failed to come to hand at the appointed time. The April number will be published on time, after which there will be no farther delay. The publishers hope this statement will be satisfactory to the friends of the RECORD, especially as the delay will very materially enhance the value of the journal.

The publishers are determined to eradicate the idea from the minds of the profession South that medical journals cannot be sustained, by giving them one which in all things, mechanical and otherwise, shall be second to no other in the Union.

If the publishers are willing to do so much for the advancement of an honored profession, it is but reasonable to hope that the profession will second their efforts, and make the RECORD the visitant of every physician within the limits of the South. Let every friend of this journal, and of an enterprise seeking the best interests of medicine and humanity, exert himself to give it a circulation commensurate with so noble and important an object. Let each subscriber become an agent, and if he can do no more, at least secure one *bona fide* subscriber.

All old subscribers will please send their subscription ($3.00) to the publishers as soon as possible. All responsible new subscribers will, if they desire it, be allowed (as old subscribers) from thirty to ninety days to cash subscriptions. They will please state, when ordering the RECORD, what time they will remit the money.

As the publishers are not acquainted with the writing of subscribers, they will do them a great favor to write legibly and plainly their names, State and county, and the post-office to which they wish the journal sent. Please remember this, and govern yourselves accordingly.

SOUTHERN PUBLISHING CO.

### TO DELINQUENTS.

---

Those of our subscribers, many of whom for the years 1873 and 1874 have failed to pay for the RECORD, we address them a few words.

We feel that some of the very best men, through negligence, have failed to pay us for a Journal sent them at our expense; and as we have been giving the Profession a Journal without compensation, it bears hard upon us if subscribers fail to pay. We are compelled to pay our publisher at great sacrifices. Money at 3 and 5 per cent. per month very soon consumes the amount. Appeals and duns by mail cost too much, and if our delinquent friends fail to see the injustice they do us, as well as the justice of our demand upon them for the small amount they owe us, we shall be compelled to forward accounts for collection with expenses added.

We are speaking to friends, all of whom are equally interested in the Journal, but neglect a plain duty both to us and themselves. If they pay us—as they will—they cannot now reimburse us for the outlay made by us in their behalf. Pay up, friends, and pay at once; ease your consciences and our pockets.

All friends—subscribers for the years mentioned—who have recently paid, have been properly credited; and as we do not wish to acknowledge dues for those years in the Journal, we will shortly forward printed receipts.

Friends remitting their dues, will do so by Check, Registered Letter, or Postal Order, which will be a guarantee of its arrival.

We will expect to hear from our good-hearted but neglectful subscribers within the next 30 days.

THOS. S. POWELL,
WM. T. GOLDSMITH.

